# Choroidal structural changes following vitrectomy performed with phacoemulsification in unilateral idiopathic epiretinal membrane

**DOI:** 10.1186/s12886-023-02803-w

**Published:** 2023-02-06

**Authors:** Yan Liu, Jia Ying Zhang, Xia Ding, Fang Lin He, Lin Na Lu, Yao Fu

**Affiliations:** 1 Department of Ophthalmology, Ninth People’s Hospital, Shanghai Jiao Tong University School of Medicine, 639 Zhizaoju Road, Shanghai, 200011 China; 2 Shanghai Key Laboratory of Orbital Diseases and Ocular Oncology, 639 Zhizaoju Road, Shanghai, 200011 China

**Keywords:** Epiretinal membrane, Choroid, Choroidal vascularity index, Vitrectomy, Phacoemulsification

## Abstract

**Background:**

This study aims to determine the influence of vitrectomy combined with macular epiretinal membrane dissection and internal limiting membrane (ILM) peeling and phacoemulsification on choroidal vasculature in patients with unilateral idiopathic epiretinal membrane (IERM) and concurrent cataract using optical coherence tomography (OCT).

**Methods:**

This retrospective study included 26 eyes of 26 patients (8 males and 18 females) with unilateral IERM without vitreomacular traction (VMT) (group 1) and the patients’ fellow eyes (*n* = 26, group 2). Three-port 25-G pars plana vitrectomy (PPV) combined with macular epiretinal membrane dissection and ILM peeling and phacoemulsification was performed on all patients. The comprehensive ophthalmologic examinations of all patients involved OCT measurements at every visit before and after surgery, and the choroidal thickness (CT), central macular thickness (CMT) and choroidal vascularity index (CVI) were calculated.

**Results:**

The mean age of the IERM patients was 66.58 ± 7.06 years. Postoperatively, best corrected visual acuity (BCVA) was significantly greater than baseline (*P* = 0.023). The CVI of the IERM eyes was significantly lower (*P* < 0.01) than that of the fellow eyes at baseline. The subfoveal CT in the IERM eyes was lower than that in the fellow eyes (*P* = 0.023), but there was, no significant difference in the average CT between the two groups at baseline (*P* = 0.071). In eyes with IERM, the CVI significantly increased at 1 week, 1 month (*P* < 0.001), and 3 months (*P* = 0.049) postoperatively, the subfoveal CT was markedly thickened 1 month after surgery (*P* = 0.001), the temporal 3 mm and nasal CT significantly increased at 1 week and 1 month postoperatively (*P* = 0.041, *P* = 0.022 for temporal 3 mm; *P* < 0.001, *P* = 0.047 for nasal 1.5 mm; *P* = 0.01, *P* = 0.001 for nasal 3 mm), and only the temporal 3 mm CT increased significantly at 3 months postoperatively (*P* = 0.017). The baseline CMT of the IERM eyes was significantly thicker than that of the fellow eyes (*P* < 0.001). CMT significantly decreased at 3 months postoperatively in IERM eyes(*P* = 0.033).

**Conclusions:**

The increase in the CVI in the IERM eyes without VMT after combined PPV with ILM peeling and phacoemulsification persists for at least 3 months.

## Background

Epiretinal membrane (ERM) is one of the most common maculopathies, which occurs due to the proliferation of fibrocellular tissue and develops on the surface of the internal limiting membrane (ILM). The incidence of idiopathic ERM (IERM) is 2.2–18.5% in the general population and higher in the older age group. The pathogenic mechanism has not been fully elucidated, and it may be idiopathic or secondary due to other retinal diseases [1].

The choroid mainly consists of blood vessels and surrounding stromal tissue and lies beneath the retinal pigment epithelium. It has an abundant blood supply and is considered a vital source of nutrition for the foveal avascular zone; therefore, normal choroidal vasculature is essential for maintaining normal retinal physiology [2–4]. Macular diseases, such as central serous chorioretinopathy, macular hole, and polypoidal choroidal vasculopathy, are associated with changes in choroidal thickness [5]. Previous studies revealed the reduction and normalization of choroidal thickness after vitrectomy, suggesting some relationship between choroidal thickness and structure and the presence of idiopathic ERMs. Therefore, it was hypothesized that changes in retinal blood flow led to a thickening of the choroid, which provided more nutrients and oxygen to the retina [6]. Vitrectomy combined with dye-assisted ILM peeling is the standard treatment for symptomatic IERM, with combined vitrectomy with cataract extraction usually performed in older people with concurrent cataracts to enhance postoperative visual rehabilitation [7, 8]. However, the effect of combined vitrectomy and cataract extraction on the choroidal vasculature is not well understood but may be related to the postoperative visual prognosis, because of the critical nourishment effect of the choroid to the photoreceptors and retinal pigment epithelium [9].

In the study, OCT was performed to quantitatively measure the choroidal thickness (CT) and choroidal vascularity index (CVI) to determine the influence of pars plana vitrectomy (PPV) performed with phacoemulsification on the choroidal vasculature in patients with IERM.

## Methods

### Study subjects

This was a retrospective study of 26 patients diagnoses with unilateral idiopathic ERM and a concurrent cataract. Group 1 included 26 eyes of IERM eyes and group 2 included these patients’ fellow eyes. The ophthalmologic examination included intraocular pressure (IOP) measured using noncontact tonometry (Topcon CT-1P, Topcon Corporation, Tokyo, Japan), best-corrected visual acuity (BCVA), axial length (AL) measured using IOL-Master 500(Carl Zeiss Meditec, Jena,Germany) and a fundal examination using a 90 diopter (D) indirect lens.

The study adhered to the tenets of the Declaration of Helsinki and was approved by the Ethical Review Committee of the 9th People’s Hospital. Patients were recruited consecutively from April 2020 to November 2021 from the Ophthalmology Department of the 9th People’s Hospital. All patients provided written informed consent.

The inclusion criteria were the presence of IERM in only one eye with a concurrent cataract and > 50 years of age. The exclusion criteria were previous intraocular surgery, ocular trauma, cataract grading≥NO4/NC4 or C4 or P2 according to the Lens Opacities Classification System III [10], high myopia (spherical equivalent of − 6.0 diopter or less), hyperopia (spherical equivalent of 2.0 diopter or more), an AL of more than 26 mm, other macular diseases, diabetic retinopathy, retinal vein occlusions, glaucoma, or uveitis, uncontrolled hypertension, and poor-quality OCT images.

### Optical coherence tomography data acquisition and processing

All study subjects were imaged using the spectral domain OCT (Spectralis OCT, Heidelberg Engineering, Heidelberg, Germany) at baseline and 1 week, 1month, and 3 months postoperatively, and the scans were tracked. Scanned images with a signal strength index of more than 25 and no residual motion artifacts were saved for further analysis. One 9 mm scan image from each follow-up visit was selected horizontally passing through the fovea for each patient.

**Fig. 1:**
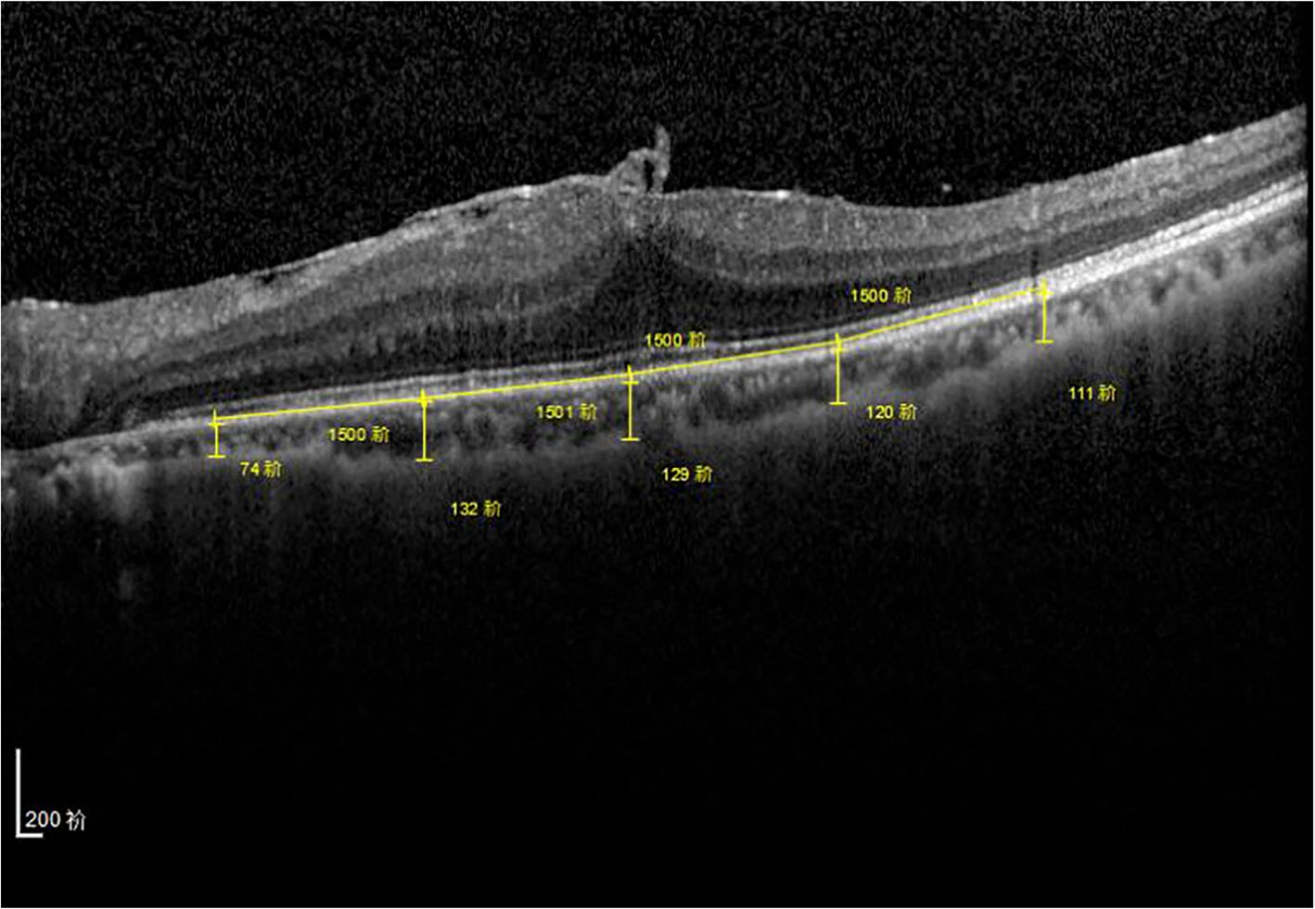
Choroidal thickness measurements were manually measured at the fovea and 1.5 and 3 mm nasal and temporal to the fovea

IERMs were classified according to the 4-grade spectral domain OCT staging system by Govetto et al. [11]. The distance from the outer border of the retinal pigment epithelium to the inner border of the sclera as the choroidal thickness measurements was manually measured at the fovea and 1.5 and 3 mm nasal and temporal to the fovea (Fig. 1). Central macular thickness (CMT) was assessed by manually measuring the distance between the vitreoretinal surface and the outer aspect of the retinal pigment epithelium (RPE)/Bruch’s membrane complex at the foveal center. All images were directly measured by an independent investigator.

### Image binarization

**Fig. 2:**
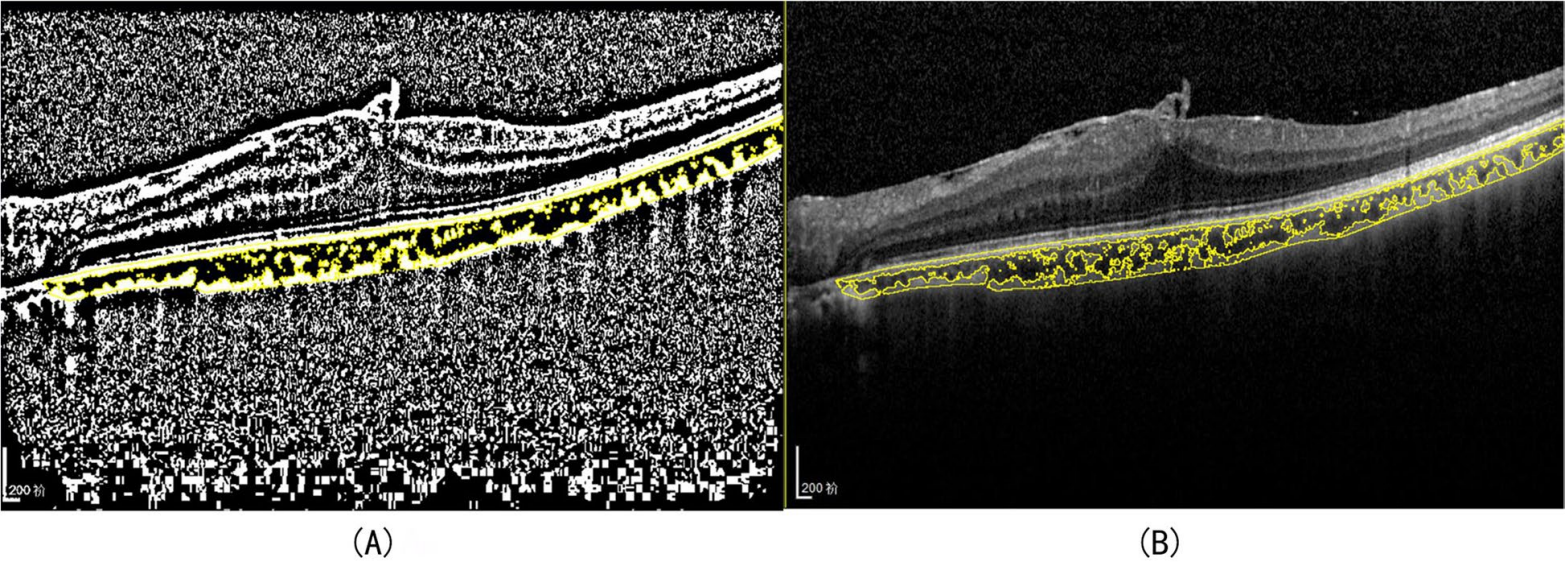
Image binarization. **A** TCA was selected, and the image was adjusted by the auto-local threshold tool. The dark pixels represented the LA and the light pixels represented SA. **B** An overlay image of the ROI of the binarized segment on the original OCT scan

Image binarization was performed using Image J (version 1.52a, http://imagej.nih.gov/ij/) as previously described with modifications [12, 13]. Briefly, one 9 mm scan image was selected, and the polygon tool was used to manually select the total choroidal area (TCA) and added to the region of interest ROI manager. Next, the original image was converted to 8 bits and adjusted through the Niblack auto local threshold. Then, the binarized image was converted back to the RGB image, and the luminal area (LA) was determined by applying the threshold tool. Dark pixels were considered LA, and the CVI was defined as the ratio of LA to TCA (Fig. 2).

### Pars plana vitrectomy

The patients underwent standard three-port 25-G PPV (Constellation Vision System; Alcon Laboratories, Inc., Fort Worth, TX, USA) combined with ERM and ILM peeling. The ILM was stained with 0.05% indocyanine green (ICG) solution and removed after triamcinolone-assisted ERM peeling. Phacoemulsification was performed before vitrectomy. Tobramycin dexamethasone eyedrops were applied topically after the operation.

### Statistical analysis

The statistical analyses were conducted using the GraphPad Prism 7.0 software (GraphPad Software, CA). Continuous values are presented as mean ± standard deviation (SD). A repeated-measures general linear model was used to compare preoperative and postoperative measurements. A paired t-test was used to compare the data of the affected eyes and fellow eyes. The significance level was set at *P* < 0.05.

## Results

This study involved a total of 52 eyes of 26 patients (8 males and 18 females), including 26 IERM eyes and 26 fellow eyes, and the patient clinical data are demonstrated in Table 1. Two patients had diabetes without diabetic retinopathy (DR). None of the patients had posterior capsular rupture during phacoemulsification. The IERM eyes did not differ significantly from the fellow eyes in terms of IOP or AL at baseline. According to the ERM staging system by Govetto et al., 8 patients (30.8%) were graded as stage 2, 14 patients (53.8%) were stage 3 and 4 patients (15.4%) were stage 4.

**Table 1:** Demographic and clinical characteristics of the study populations

		ERM eyes (*n* = 26)	Fellow eyes (*n* = 26)	*P* value
Age, years (mean ± SD)		66.58 ± 7.06		/
Gender (male/female)		8/18		/
BCVA, log MAR (mean ± SD)	baseline	0.52 ± 0.33	0.17 ± 0.17	< 0.001
	3 Mo Postop	0.35 ± 0.17	/	/
IOP, mmHg (mean ± SD)	baseline	16.15 ± 2.13	16.2 ± 3.04	0.95
	1 Wk Postop	13 ± 4.8	16.05 ± 2.81	0.002
	1 Mo Postop	16.04 ± 2.93	15.87 ± 3.31	0.74
	3 Mo Postop	16.23 ± 2.42	16.02 ± 2.95	0.681
axial length, mm (mean ± SD)		23.26 ± 0.84	23.44 ± 0.83	0.364

The BCVA significantly improved 3 months postoperatively (*P* = 0.023 compared to baseline). The IOP and AL did not differ significantly between the IERM and fellow eyes. The mean baseline IOP of IERM eyes and fellow eyes was 16.15 ± 2.13 mmHg and 16.2 ± 3.04 mmHg, respectively. The IOP of the IERM eyes significantly decreased 1 week postoperatively (*P* = 0.002) but was not significantly different from the preoperative values 1 month and 3 months postoperatively (*P* = 0.74, 0.681).

### CVI

**Fig. 3:**
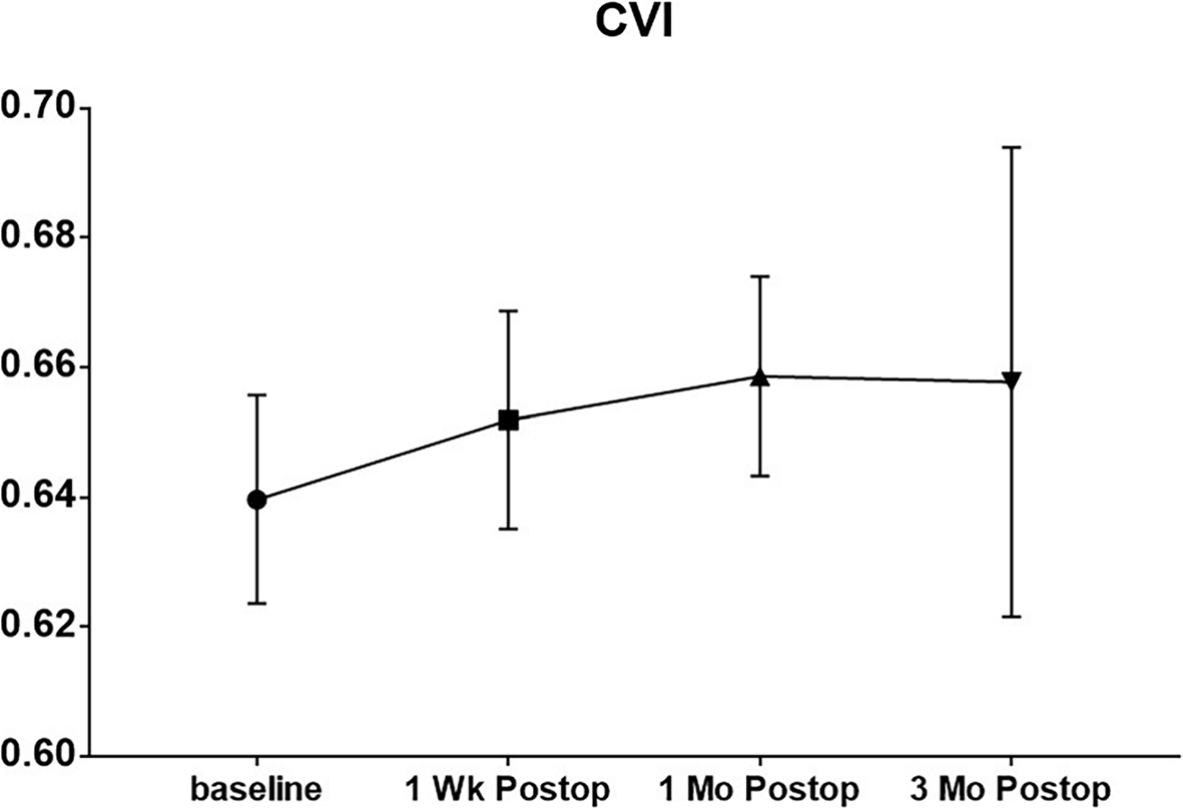
The choroidal vascularity index of the IERM patients at the four visits (mean ± SD)

The baseline CVI in the IERM eyes was significantly lower (*P* = 0.011) than that in the fellow eyes (Table 2) and significantly increased at 1 week, 1 month and 3 months postoperatively. (*P* = 0.007, *P* < 0.001, *P* = 0.049 for 1 week, 1 month, 3 months postoperatively compared with baseline respectively) (Fig. 3).

**Table 2:** The CT, CVI and CMT of the IERM eyes and the fellow eyes at baseline. (mean ± SD)

			ERM eyes	Fellow eyes	*P* value
CT (μm)	Fovea		205.2 ± 99.29	220.6 ± 113.2	0.023
	Temporal	1.5 mm	213.7 ± 89.26	211 ± 87.65	0.812
		3 mm	186.5 ± 87.25	197.6 ± 80.38	0.458
	Nasal	1.5 mm	163 ± 75.7	179.9 ± 71.24	0.036
		3 mm	103.8 ± 42.9	116 ± 44.2	0.025
	Average		174.4 ± 70.83	185.4 ± 71.69	0.071
CMT (μm)			409.3 ± 128.2	238 ± 61.78	< 0.001
CVI(%)			63.97 ± 1.6	65.29 ± 2.34	0.011

### CT

The baseline subfoveal and nasal CT of the IERM eyes was significantly thinner than that of the fellow eyes (*P* = 0.023, 0.036, 0.025 for CT values of the subfovea, 1.5 and 3.0 mm nasal to the fovea, respectively), although there was no difference in average CT of the macula(*P* = 0.071) (Table 2). Subfoveal CT significantly increased at 1 month postoperatively in IERM eyes; the temporal 3 mm and nasal CT significantly increased at 1 week and 1 month postoperatively, but only the temporal 3 mm CT values increased at 3 months postoperatively (Table 3). Subfoveal CT, temporal 1.5 mm CT and nasal 1.5 mm CT of 3 months after surgery decreased significantly when compared with the values of 1 week postoperatively(*P* = 0.008, *P* = 0.030, *P* = 0.0059, respectively).

**Table 3:** The preoperative and postoperative CT and CMT of the IERM eyes at the four visits. (mean ± SD)

		Baseline	Postop					
			1 Wk Postop	*P*-value	1 Mo Postop	*P*-value	3 Mo Postop	*P*-value
Foveal CT (μm)		205.2 ± 99.29	221 ± 100.2	0.123	233.9 ± 104.6	0.006	216.4 ± 98.38	0.446
Temporal CT (μm)	1.5 mm	213.7 ± 89.26	230.8 ± 81.52	0.144	229.5 ± 67.26	0.311	219.8 ± 85.2	0.813
	3 mm	186.5 ± 87.25	200 ± 75.84	0.041	206.4 ± 75.5	0.022	197.5 ± 79.91	0.017
Nasal CT (μm)	1.5 mm	163 ± 75.7	180.2 ± 81.79	< 0.001	185.7 ± 70.53	0.047	166.4 ± 66.59	0.97
	3 mm	103.8 ± 42.9	121.7 ± 46.75	0.01	122.8 ± 34.43	0.001	112 ± 35.55	0.453
CMT (μm)		409.3 ± 128.2	392.1 ± 108.5	0.655	385.1 ± 103.6	0.353	358.9 ± 92.19	0.033

### CMT

The baseline CMT of the IERM eyes was significantly thicker than that of the fellow eyes (*P* < 0.001) (Table 2). CMT significantly decreased at 3 months postoperatively in IERM eyes(*P* = 0.033) (Table 3).

## Discussion

The CVI represents the proportion of the lumens of choroidal vasculature in the choroid and is considered a marker of choroid vascular health [14, 15] as well as a relatively stable and consistent indicator of choroidal disease progression. It has been frequently used in evaluating the choroidal vascular structure changes in various ocular diseases such as uveitis or diabetic retinopathy [12, 16, 17].

It has been reported that the CVI and/or CT increased after cataract surgery, which lasted at least 3 months [17], whereas the CVI and/or CT decreased after PPV. Thus, there is a possibility that phacoemulsification and vitrectomy have opposite effects on the choroidal vessels. Understanding the changes in the choroid after combined vitrectomy with phacoemulsification can help predict the visual prognosis of ERM patients with cataract after the combined operations. Nevertheless, the influence of combined surgeries on choroidal vasculature is not well understood in this population, so this study aimed to investigate the effects of combined operations on the choroidal vasculature.

The changes in CT and CVI were measured before and after phacovitrectomy to explore the choroidal vasculature changes in IERM and the influence of surgery, showing that the CVI of the IERM patients increased significantly within 3 months after phacovitrectomy surgery, which suggests choroidal vessels dilatation. At the same time, the CT also increased significantly in some areas of the macula, in line with CT changes after cataract surgery. Prolonged inflammatory reactions have been described after cataract surgery [18] with proinflammatory cytokines expressed in the choroid following cataract surgery, and there is evidence of upregulation of IL-1β and CCL2 gene and protein expression in the choroid [19]. The vitrectomy also causes inflammation as part of the healing process, with increased cytokines such as TGF-β1, IL-8, and IL-6 in the aqueous humor [20]. In addition, the reduced IOP is likely to cause increased CT in the early period following phacoemulsification performed with or without vitrectomy due to increased ocular perfusion pressure [21, 22]. The present study demonstrated that the CT/CVI increased in the early postoperative period, which is similar to that reported in ERM and MH patients undergoing vitrectomy [21, 23]. Therefore, the upregulation of proinflammatory cytokines and the decreased IOP after surgery might cause an increased CVI/CT after combined vitrectomy. The decreased IOP might increase the CVI/CT in the early postoperative period, which lasts at least 1 week postoperatively, and the inflammation-induced increase in CVI/CT might last at least 3 months.

Moreover, recurrent ERM is also a process of fibrocellular proliferation caused by excessive collagen production by hyalocytes, RPE cells, or retinal glial cells, which might remain after surgery or migrate to the surface of the retina because of surgical trauma. Though the cause of ERM recurrence is unclear, greater surgical trauma and subsequent postoperative inflammation might increase the recurrence rate [24]. Whether the increased surgical trauma and inflammatory response caused by combined surgery increase the recurrence rate compared with non-combined surgery may be worthy of further studies.

The pathological mechanism of IERM has not been fully elucidated, but posterior vitreous detachment (PVD) is considered an important pathogenic factor. Anteroposterior (AP) traction promotes the expression of growth factors, including vascular endothelial growth factor (VEGF) and basic fibroblast growth factor (bFGF) in the retina [25, 26]. In our study, the 26 patients with unilateral IERM without VMT lacked mechanical AP traction, but the IERM eyes did not have a greater CT than the fellow eyes. Although the average CT of the macula in ERM eyes was not significantly different from that of the fellow eyes, the subfoveal and nasal CT values of ERM eyes were respectively thinner than that of fellow eyes at baseline. Interestingly, CVI was lower at baseline in the ERM eyes than in the fellow eyes, which might imply the possible involvement of abnormal choroidal vessels in the pathogenesis of IERM, especially unilateral IERM. Kang et al. found that choroidal thickness was greater in IERM eyes with than without VMT, suggesting that VMT could lead to choroid thickening, but they did not compare the results with fellow eyes [27]. Other studies did not observe any difference in the CT between the fellow eyes [28] or a significantly higher CT in IERM eyes than in fellow eyes [29]. However, the presence or absence of VMT was not described in these studies, and not all patients were unilateral, which may be the reason for the inconsistency in results. Therefore, further larger studies are needed with more precise grouping.

It was reported that ERM could influence the choroid microvasculature through ERM-associated traction [30]. In this study, CMT decreased significantly postoperatively compared with baseline 3 months after ERM-ILM removal. The release of mechanical stretching mainly led to a decrease in thickness of retina. As mentioned above, CT increased after surgery, whereas CT subsequently decreased gradually within 3 months postoperatively, which might be due to the combined effect of reduced mechanical stretching and decreased level of VEGF and inflammatory cytokines as time went on after surgery.

This study has some limitations. First, the relatively small sample size, but the patients had a consistent diagnosis, homogeneous ethnicity, and underwent the same surgery method, thereby reducing some confounding effects. A second limitation is the short period of follow-up of 3 months, so a long-term follow-up should be conducted with a larger sample.

In conclusion, the present study revealed increased CVI and thickened CT following phacoemulsification performed with vitrectomy in eyes with unilateral idiopathic epiretinal membrane without VMT, suggesting that the choroidal vessels dilate due to postoperative inflammation. However, whether the increased postoperative inflammation could be a potential risk of recurrence requires further investigation. Future studies should involve a long-term follow-up to determine how long the increase in CVI/CT and the possible latent postoperative inflammation following phacoemulsification will last after surgery, as well as the possible effect on visual prognosis and ERM recurrence in this population.

## Data Availability

The datasets used and/or analyzed during the current study are available from the corresponding author on reasonable request.

## Abbreviations

ILM: Internal limiting membrane
IERM: Idiopathic epiretinal membrane
OCT: Optical coherence tomography
VMT: Vitreomacular traction
CT: Choroidal thickness
CVI: Choroidal vascularity index
BCVA: Best corrected visual acuity
ERM: Epiretinal membrane
LA: Vascular luminal area
TCA: Total choroidal area
PPV: Pars plana vitrectomy
IOP: Intraocular pressure
AL: Axial length
CMT: Central macular thickness
SD: Standard deviation
DR: Diabetic retinopathy
VEGF: Vascular endothelial growth factor
bFGF: Basic fibroblast growth factor
CSC: Central serous chorioretinopathy
